# AI Technologies, Privacy, and Security

**DOI:** 10.3389/frai.2022.826737

**Published:** 2022-04-13

**Authors:** David Elliott, Eldon Soifer

**Affiliations:** Department of Philosophy and Classics, University of Regina, Regina, SK, Canada

**Keywords:** ethics, privacy, security, artificial intelligence, autonomy, semantic understanding

## Abstract

Privacy remains one of the most recurrent concerns that people have about AI technologies. The meaning of the concept of “privacy” has proven to be fairly elusive. Accordingly, the concerns people have about privacy are often vague and ill-formed, which makes it correspondingly difficult to address these concerns, and to explain the ways in which AI technologies do or do not pose threats to people's interests. In this article, we draw attention to some important distinctions that are frequently overlooked, and spell out their implications for concerns about the threats that AI-related technology poses for privacy. We argue that, when people express concerns about privacy in relation to AI technologies, they are usually referring to security interests rather than interests in privacy *per se*. Nevertheless, we argue that focusing primarily on security interests misses the importance that interests in privacy *per se* have through their contribution to autonomy and the development of our identities. Improving insight about these issues can make it easier for the developers of AI technologies to provide explanations for users about what interests are and are not at stake through the use of AI systems.

## Introduction

Among the most prominent concerns people have about AI technologies are those regarding privacy. “Privacy,” however, has proven to be a fairly elusive concept, as indicated by the lack of any clear consensus about its nature within the philosophical or legal literature. In the philosophical literature, it has been commonly described as a “concept in disarray” (Solove, 2008), and, even more dramatically as evocative of entering an “unknown swamp” (Inness, 1992). Indeed, some philosophers have come to believe that privacy does not represent a single, coherent concept at all (Thomson, 1975; Solove, 2008). Accordingly, the concerns people have had about privacy are often vague and ill-formed, which makes it correspondingly difficult to address these concerns, and explain the ways in which AI technologies do or do not pose threats to people's interests. In this article, we will draw attention to some important distinctions that are frequently overlooked, and spell out their implications for concerns about the threats that AI-related technology poses for privacy.

It is common to assume that privacy is all about being able to control information about oneself, generally in the form of having a right to prevent others from obtaining or using information about a person without that person's consent. Important criticisms have been raised against such a “control” account of privacy (Thomson, 1975; Macnish, 2018), and although we will not enter fully into this dispute, we do argue that it is a mistake simply to equate a lack of control with a lack of privacy. Understanding why this is a mistake requires drawing attention to some often-ignored features of the concept of privacy, including the significant role played by beings capable of semantic understanding (Soifer and Elliott, 2014; Marmor, 2015; Macnish, 2020).

Ethical discussions about artificial intelligence and privacy often focus on questions about how a “right to privacy” of the sort outlined above can be balanced with the benefits of allowing companies unfettered access to users' information (such as the ability to personalize services for clients, and corporate profitability, with corresponding benefits to the overall economy), and about what scheme of regulation is likely to provide the best outcome overall. It is not the point of this essay to engage with those discussions directly. Rather, it will focus on a prior question, about why this sort of user control should be considered valuable in the first place. The standard answer would be that it protects various other interests that people have, in the sense that, if people obtain information about a person without that person's consent, they might use that information to harm the person in any of a variety of ways (e.g., denying a loan, identity theft, etc.). There undoubtedly are such interests at stake with regard to “private” information, and we refer to those interests as “security interests.” However, we want to contend that there is another important interest at stake here as well, which in fact is more properly described as an interest in privacy than the security interests. Correspondingly, we refer to this other interest as an interest in privacy *per se*. We argue that this other, largely neglected, interest has not only constituted a major part of how privacy has traditionally been understood, but is also of significant value because of its connection with human autonomy. However, discussions of the relationship between artificial intelligence and privacy have largely ignored this other interest underlying privacy, and this essay seeks to redress that shortcoming by exploring the interaction between artificial intelligence and privacy understood in this way.

## Conceptual Matters

### Privacy and Other Interests

One of the challenges that faces any discussion of privacy is that privacy interests can easily be confused with a range of other interests or moral concerns, and it is important to try to sort some of this out conceptually.

Privacy is often associated with the acquisition of information about people. The term is not always used in this way—for example, a person making so much noise in the neighborhood that you cannot concentrate enough to read a book within your own home might be described as “intruding on your privacy,” even though their making noise does not help them acquire any information about you. In this article, however, we will focus on “informational privacy,” and ignore other possible uses of the term such as this. Similarly, although some people want to restrict the term “privacy” to the acquisition of only certain *kinds* of information about people (e.g., so-called “personal information”), we will not directly address here debates concerning the appropriate extension of the term in that sense either.

Within the context of information about people, the term “private” is sometimes used to distinguish information that is not known by others from information that is. In this sense, “private information” is whatever information people do not happen to know about other people. Note that, given that information about a person may be known by some people but not others, it might be more accurate to say that a certain bit of information is “private in regard to person A,” understood as consistent with being “not private in regard to person B.” Some people might insist that an item of information should be considered “private” if it is not known by *anyone* other than the person it is information about, or, even more strongly, if it is impossible for anyone to find out the information without the person's choosing to divulge it (Parent, 1983). We do not think these are the most useful ways in which to use the term, and are skeptical about whether privacy in the stronger sense is even possible, but we will not focus on any of these additional issues either, within the current work. It is worthwhile to note, though, in this context, an important distinction between the objective claim that a certain bit of information is not known by others, and the normative claim that it *should not* be known by others (e.g., that it would be morally wrong for others to try to acquire that information).

In this article, we will also not be much concerned with this usage of “private,” to describe whether some particular bit of information is or is not known by others. Instead, we will focus on what might be called “privacy interests”—the interests that people may have in avoiding having others acquire information about them. It may be worthwhile here to note a further distinction concerning how it is that others acquire the information. In some cases, someone may not be trying to acquire such information, and just comes upon it by chance, as it were. This could occur, say, if a piece of paper containing information a person would like to keep private blows out of the window and into the path of someone who then innocently reads it, or if people are forced to flee a fire and are thus seen in a public place in a condition they would prefer not to have been seen in, through no fault of the observer. In such cases, we would describe privacy as having been “lost.” This is to be distinguished from cases in which the person acquiring the information deliberately tried to acquire it when it was not proper to do so. In those cases, we would say privacy has been “violated.”

Most commonly, when people consider such “privacy interests,” what they have in mind are the interests people have in controlling information about themselves or preventing others from knowing things about them without their consent. There are reasons to wonder whether this might be the best way to reason about privacy (Menges, forthcoming). However, examining these reasons is not essential to our purposes here. What is important is to note that one reason people are generally concerned about control is that they are concerned that if others have certain sorts of information about us they might be able to use that information in ways that will harm us. For example, banking secrets can be used by thieves to steal money from my bank accounts, or a disgruntled customer who learns my home address might use that knowledge to come to my home and attack me or damage my property. Because cases like these involve people using information they have acquired about others in ways that harm those others, we will describe the interest people have in avoiding having this happen as “security interests.” Protection of such “security interests” constitutes an important reason people have for wanting to avoid having others acquire information about them. (When we use the word “security” here, we are referring to the security of the person, or protection of the individual against harm. This is distinct from another usage of the word to indicate security of data, meaning the prevention of unauthorized access to it.)

Notice, however, that, in the cases discussed above, the problem does not lie simply in the acquisition of information about someone. The person acquiring the information has to take some further action on the basis of that information—such as accessing my bank accounts, or attacking me in my home. This raises the possibility that a person could acquire lots of information about someone else, but never use that information to harm the other. Thus, there could be what Tony Doyle has called a “perfect voyeur” (Doyle, 2009) who observes another, but never does anything with that information that is harmful to the other. In such a case, the “security interests” of the person observed are not compromised—but that does not mean that there has been no violation of privacy. Indeed, we believe this simply reveals another sort of interest that people have in avoiding having others acquire information about them, which we call an interest in “privacy *per se*.”

### A Theory About the Nature of Privacy

We have developed a view about privacy which takes this interest in “privacy *per se*” to be central, and we believe this view can provide an excellent way of accounting for a variety of intuitions people have about the nature and value of privacy. It is not possible to provide a detailed defense of our view within the current article, however, this view or similar ones have been described and defended elsewhere (Johnson, 1989; Feldman, 1994; Velleman, 2001; Shoemaker, 2010; Soifer and Elliott, 2014; Marmor, 2015). Nevertheless, a brief sketch of how we understand the nature of privacy can be outlined here. We will then give particular scrutiny to the impact that AI can have on three specific elements of privacy, thus understood: what we will refer to as epistemic privilege, consent/control, and the “feedback loop.”

In our view, the interest in privacy *per se* stems from the fact that people care how others perceive them. Since how a person perceives another is generally grounded in what the person knows or believes about the other, this interest in having people perceive one in a certain way naturally gives rise to an interest in controlling the information about oneself that others acquire.

Throughout history, humans have been able to acquire information about others, and use it as the basis for judgments about them. It is likely that this tendency in fact had tremendous survival value. For a species that depends on being able to combine the strength of a number of individuals to defend the group against challenges, it is very important to be able to make judgments about whom one can depend on in a crisis (Dunbar, 2004). It is also useful to note in this context Jean-Jacques Rousseau's interesting (though not well-grounded historically) claim that this ability, and the corresponding interest in how others perceive one, arose as soon as humans began living together. He describes dancing as one of people's first activities together, and then says they each immediately wanted to be seen as the best dancer (Rousseau, 1990).

This capacity to acquire information has always encountered some direct limitations, however. It has certainly not been possible to observe others at all times, and thus a number of one's activities, beliefs, and tendencies have been unavailable to others in practice. That is to say, ordinary people have a number of limitations with regard to their observational and epistemic capabilities. Since people do observe their own activities and attitudes directly, however, it has standardly been the case that people know more about themselves than others do—they are epistemically privileged in this regard. There are some exceptions to this, of course. For example, parents may know and understand more about their young children than their children do of themselves. Even among adults, it is possible that a good friend, for example, might “know me better than I know myself.” Nevertheless, it has generally been the case that people have had this sort of epistemic privilege concerning information about themselves.

This epistemic privilege makes it possible for people to influence how others perceive them by controlling the information about them available to others. This is done through making decisions about which bits of information about themselves, if revealed, are likely to lead others to perceive them in ways they want to be perceived. We describe this as the process of “persona-building.” Generally, people want to be perceived in different ways by different others, and thus it would be more accurate to say that people build a number of personae, rather than a singular persona, and they achieve this at least in part by revealing different bits of information about themselves to different others. We claim that this activity of persona-building is the fundamental activity characteristic of privacy *per se*. It is important to note that this process seems to depend on epistemic privilege. If, by contrast, we imagine a situation in ordinary life where the people we are trying to present ourselves to in a certain way know everything there is to know about us, then it seems fairly clear that no such persona development is possible—and thus, on our view, no privacy is possible either.

This process of persona-building typically involves a second feature, which we will also take particular notice of in the context of AI. This is that people usually exercise considerable control over their persona creation. If people have privacy, they are the ones who are intentionally generating the personae of their choice. The person in question—the data subject—is the one who selects and presents information, often in the form of behavior, that they believe will represent the profile they have in mind to other people. Other people can then choose to accept this or not, generate their own interpretation of the information they have been given, or, among other options, ignore the persona presentation entirely.

We also believe that this practice of deciding what to reveal or conceal about oneself so as to influence others' perceptions of one has played a little-noticed but very important role in the development and exercise of autonomy. This comes about not only because the process allows people to make choices about how to present themselves, but also because people attempting to build personae generally get important feedback from how others react to their efforts. That is, usually we have the opportunity to see how our self-presentation is received by others, and adjust our understanding of ourselves or our self-presentation to others (or both). This process is vital for both self-understanding and self-development, and these in turn are crucial for the development of an autonomous self. Where no such response by others is available, the opportunities for autonomous self-development are thereby diminished. This process also highlights the significance of the embeddedness of individual autonomy within social and political contexts, and it does not depend on controversial ideas of the choosing self as existing prior to and independently of any social influences (Cohen, 2013; Mokrosinska, 2018). Whether a person's self-presentation and involvement with others results in the indifference, encouragement, engagement, or hostility of others has a significant impact on the opportunities for individual autonomy.

To illustrate, consider a person who wants others to think of them as witty. If I were such a person, I might I make remarks that I think will cause them to see me in this way. If the others do not laugh at my remarks, then I might conclude that they did not perceive me to be as witty as I wanted them to. To put this another way, they did not think that aspect of the persona I was trying to project “fit” me. I may therefore learn something about myself from this interaction—that I am not really very witty (Of course, that may not be the conclusion that I draw. I might, for example, decide instead that what I said really was witty, and thus I really am a witty person, but that the others are poor judges of true wit. But we will focus on the more common case, where the reaction of other is taken to reveal something about oneself.). We refer to this process of learning about oneself through how others react to the personae we try to project as a “feedback loop.” We believe that this “loop” plays a crucial role in the development of self-understanding, and thus in the formation of autonomous agency, and it is the third element of privacy we want to pay particular attention to within the context of AI.

It is important to stress here that in presenting this account, we are not suggesting that people have an absolute right to present themselves however they want or that unwanted observation can or should always be avoided. Rather, our position is that there is a significant value at stake in persona-building, even if it can be overridden by other compelling interests.

## The Impact of AI Systems on Key Elements of Privacy

With these remarks about the general nature of privacy in place, it is now possible to use these understandings to provide perspective on the impact of artificial intelligence on privacy concerns. We will focus on three aspects of privacy that we have noted: the notion of epistemic privilege, the element of control or consent, and the element of the feedback loop, with corresponding implications for the distinction between security interests and interests in privacy *per se*.

### Epistemic Privilege

The first aspect to be considered is the one relating to epistemic privilege. As we have seen, the interest in privacy *per se* draws on the fact that, ordinarily, people are in a position of epistemic privilege relative to others (that is, they know more about themselves than others do), and thus are able to choose which features to reveal or to conceal so as to build a desired persona. The situation is very different in regard to artificial intelligence (AI), however, particularly in conjunction with “mass surveillance.” This surveillance, as Kevin Macnish has defined it, is the “*automated* collection and processing of people's data irrespective of whether those people are liable for surveillance” (Macnish, 2020, emphasis in original). This surveillance is carried out in varying degrees and for various reasons by both governments and private companies. In addition to being automated, this data collection process is also indiscriminate—it targets virtually everyone who uses a cell-phone or an “Internet of Things” device or application. It is often covert—at least in the sense that many persons whose data is gathered may not fully know and understand its amount and/or extent. The data collection is also massive; it is much more comprehensive than the non-digital monitoring of individuals has ever been.

It may be argued that IT contexts, particularly with regard to mass surveillance, deny or nullify the epistemic privilege that underlies privacy. On-line social media—YouTube, for example—seem to “know” how to keep you watching much longer and excessively, even when you know you should have stopped long ago. And it “knows” what you are interested in—politically, personally, commercially, and so forth. More specifically, the detail and rigor of digital mass surveillance is arguably much more extensive and complete than any standard, non-digital surveillance practices.

One reason for this, of course, is the multiple sources of this data which can be relentlessly and thoroughly cross-referenced through the ubiquitous use of intelligent technologies that are now commonplace in people's everyday environments. At least two related technologies that have been developed over the last several decades in software engineering and computer science are “ubiquitous computing” and “ambient intelligence.” Ubiquitous computing involves the idea of various technologies which “weave themselves into the fabric of everyday life until they are indistinguishable from it” (Weiser, 1991, see also Weiser, 1993a,b; Weiser and Brown, 1996). Philip Brey aptly described this form of interaction with computers as a situation where computing devices “do not appear as distinct objects, but are embedded into the everyday working and living environments in an invisible unobtrusive way. They make information, media and network access constantly and transparently available” (Brey, 2005). Ambient intelligence is a broader concept (which includes ubiquitous computing) that involves networked devices that are integrated into a persistent environment. It is designed to recognize and respond to persons, anticipate their behavior, even their mental lives and desires, and then to adapt to their changing behaviors (Brey, 2005; Aarts and Wichert, 2009). Of course, this system never gets tired: it operates day and night, and perhaps in contexts in which data subjects (people) may have no significant awareness of its presence or operation. When these and similar technologies are used to surveil data subjects, gather massive amounts of data about them, then process this information and establish profiles about them, it is not surprising that these systems provide more information about their data subjects than the subjects may know about themselves. Furthermore, this data is routinely analyzed in statistical, inductive terms, and in a strict, comprehensive way that no individual would normally be able to perform by themselves. It seems clear to many observers that such technology has a greater epistemic—or at least an informational—privilege with regard to data subjects than they have of themselves. Thus, it is not difficult to imagine that such technologies are now—or could possibly be in the near future—in a nearly god-like situation in which the automated data-gathering system “knows” almost everything there is to know about its data subjects (Elliott and Soifer, 2017). We will address below the extent to which any of this involves a violation of people's privacy.

### Consent and Control

With regard to the second aspect of the concept of privacy we have been considering, as we have seen, in ordinary inter-personal interactions, people can engage in the process of persona-building by exercising control over which bits of information about themselves they want to reveal to others, and which they want to conceal. This takes place against a set of normative restrictions on people trying to acquire certain sorts of information about others without their consent (e.g., reading someone else's diary, or watching them through the windows). Automated data-gathering systems do not appear to leave any comparable allowances for control or consent. Automated data-gathering systems seem to completely ignore what the data subject intends for herself, and it simply comes to its own conclusions. The automated, AI enabled data-gathering system is the profile maker and generator—not the data subject.

In fact, if this system's ubiquitous features are operative, the data subject has very little conscious, intentional input—if any at all—into its entire data-gathering, processing, and profiling activities. Furthermore, as noted in the previous section, the data subject may not even have any awareness at all that it is actively collecting data and profiling them. This, again, seems quite dissimilar to ordinary relationships that people have in public life. In these situations, people themselves select when and how they want to be profiled, when they expect someone else to be making profiling judgments about them, and when they do not want such profiling to occur. And people, again in ordinary public circumstances, can do things to control or regulate the perceptions of others by exercising selective control over the information that they present to other people.

It must be acknowledged that, in ordinary inter-personal contexts, this control has its limitations. First, people do not always choose whether others profile them or not: there are many cases in which people wish other were not scrutinizing them, but feel they cannot escape others' gaze, perhaps because they have to engage in activities in the public realm. In fact, people we might also want others to take notice of them, and be frustrated that others are not “profiling them” when they want them to be doing so—e.g., when others are ignoring them, or just not attentive at the moment they wanted them to be. Secondly, a person may intend to remain completely anonymous when they go somewhere in public, but then a judgmental, teetotalling acquaintance notices them going into the liquor store. Also, while people may have some measure of control over the personal information that they provide to others, they have much less control over how this information is received or interpreted by others.

Despite these limitations in the ordinary case, however, people still seem to have considerable control over the information about them that is made available to others, and the conditions under which it is presented. Furthermore, there are well-established social and legal norms that regulate ordinary social and public contexts, that serve to protect people's ability to control these matters. Although data subjects do have some legal protections in most jurisdictions, it still seems to us that individuals have much less control, of the sort we have described above, in the context of automated AI data-gathering systems than in ordinary, inter-personal relationships.

### The Feedback Loop, Autonomy, and the Interests in Security vs. Privacy *per se*

In some ways, modern technology seems to enhance people's ability to develop their personae. For one thing, social media makes it possible for individuals to communicate with large numbers of other people. This means that people who build personae through social media are not merely generating personae vis-à-vis a few other individuals, but to large numbers of others. This suggests a net gain in overall capacity for persona-building (This is a very general effect, but it can perhaps be seen most clearly through the use that has been made of social media options by politicians. In the United States, Barack Obama was the first to make effective use of these possibilities, but Donald Trump brought the possibilities to new heights—or lows). Moreover, the mere fact that the communication is through electronic devices rather than being face-to-face means that social media make it easier for people to conceal some facts about themselves, which is helpful for constructing one's personae. Indeed, it facilitates the possibility of developing personae that have virtually no connection with the person whose personae they are—either in a way that is transparent to all, as in role-playing games, or in ways that are designed to deceive others, such as when 40-year-olds present themselves as teenagers on line (As noted above, we do not claim that all instances of persona-building are morally worthwhile. In noting the importance of persona-building for autonomous development, we note this is not an absolute value, and it may be overridden by other values.). However, these are simply ways in which technologies present tools that people can use in the process of trying to shape how others will see them. That is to say, they are merely ways to enhance what people were capable of doing in any case—they bring about quantitative change, not qualitative.

If digital technologies only provided these ways in which people could present themselves, this fact would certainly have some impact on privacy *per se*, as we have defined it. Although people have always had the ability to portray themselves in authentic ways and also the ability to mislead others, both of these would be augmented. There is considerable room for debate here about whether the benefits of this change would outweigh the costs. However, rather than entering into that debate here, we want to move on to discuss other features commonly associated with these technologies, which seem to have a significant impact on privacy.

We have already seen that modern technologies can gather huge amounts of information about people, undermining the traditional epistemic privilege people have enjoyed about themselves, and that they have also limited the extent to which the acquisition of information about people is constrained by their consent or is subject to their control. However, in the ordinary case, the concern about being able to conceal some information, at one's own discretion, is seen as being in service of a more foundational interest—the concern about how other people will perceive one. One major question that remains involves whether the loss of epistemic privilege and control occasioned by modern technologies has an impact on this interest in how we are going to be perceived.

There is one obvious way in which the technological acquisition of great quantities of information about a person might have an effect on how that person is perceived by others: those others might be able to access that information, and thus come into possession of the sorts of information about the person that can form the basis of their perceptions. If this happens, the interest in privacy *per se* seems to have been compromised, because the object of observation has lost the capacity to shape how that other perceives them.

There are a couple of points that need to be made, however, that may seem to mitigate the problem here. The first of these is that, in many cases, even if someone obtains the information about a person from the technological observer, there is a very good chance that this information will be anonymous. The person acquiring the information may come to know that there is someone with certain characteristics, and may have an opinion about people who have those characteristics, but may not really be able to link those particular characteristics to a specific individual in any meaningful way. In that case, it would probably not be accurate to say that the person who obtains the information is forming a perception of the person whose information has been gathered in a way that threatens the interest underlying privacy.

To illustrate the point here, it might be useful to consider an analogy. Suppose a modest woman in a Western society realizes she has to go somewhere where she will be seen by others, and that the only covering she has available is a small towel that will not cover all of the parts that people in Western societies standardly consider “private parts.” It may be that the best way to protect her privacy, in such a case, would be to wrap the towel around her head so that she will not be recognized. None of the parts she would normally want to protect against others' gaze would be protected, but the idea is that people who observed those parts would not be able to associate them with her in particular, and thus the information would remain private in a sense.

Beside the fact that information gained *via* AI systems is likely to be anonymous, there is another feature that can tend to mitigate the worry that the gathering of this information might lead to perceptions of people of a sort they do not want. This is that it is not inevitable that somebody will access that information in the first place. In ordinary cases, we have claimed, the interest in privacy *per se* is grounded on the fact that people care how they are perceived by others, and thus care what information others acquire that might form the basis of such perceptions. If information about a person is gathered through mass surveillance, and then stored digitally somewhere, but never accessed, then it seems that information cannot become the basis of anyone's unwanted perception of that person. If the information is stored electronically somewhere but never accessed by anyone, then people do not have the information, and thus cannot be basing any perception on it at all.

Given this, it seems that the collection of massive amounts of information about people might not actually compromise their privacy at all, if we understand privacy to consist of what we have been calling privacy *per se*. So long as there is a possibility that a person will access the information about people that is being stored, this technology could be understood as creating a risk that privacy will be violated, but it does not itself violate that privacy (Soifer and Elliott, 2014; Macnish, 2020). It follows that people who express concern about their privacy when information is gathered about them are largely mistaken in forming this concern.

This is not to say, however, that these people are wrong to be concerned about the gathering of such information. The gathering of it may be harmful in ways other than violating privacy. For example, information gathered about a person might be fed into an algorithm that calculates a person's credit rating, and thus some of the information gathered might, for example, lead to someone being denied a loan or a mortgage. Or, to use an example discussed by Macnish, a robot might gather information about somebody's having contravened a law and might be programmed to initiate legal action against a person in such circumstances, even without any person forming an unwanted perception of the individual (Macnish, 2020). In other words, the acquisition of this information might be responsible for considerable damage to one's security interests, even without there being any damage to one's interest in privacy *per se*. Understanding this shows the importance of drawing the distinction we noted earlier between a security interest and an interest in privacy *per se*, and could also be very important for explaining to individuals what is and what is not at stake as a result of mass surveillance.

It is possible to object to this line of reasoning on the basis that there is after all damage to the person's privacy when digital information is gathered about them, even if that is not accessed by any person, because the artificial intelligence involved is generating a profile of the person, which, it might be argued, is essentially equivalent to the sort of perception a person might form. The strength of this objection will hinge on the extent to which the profile produced by AI really is analogous to the perception a human being would normally have of another human being. We believe that it is not a very close analogy at all.

For comparison, it might be useful here to consider the perception of an individual by a different sort of observer (Soifer and Elliott, 2014; Macnish, 2020). Suppose, for example, that, as a person is going about their usual daily activities, they are being observed by the neighbor's cat (Soifer and Elliott, 2014). Although some people might be made somewhat self-conscious by the observation of a cat, we think most people would agree, and rightly so, that this observation does not pose the same sort of threat to one's privacy as observation by the cat's owner would. The question, then, is whether observation by AI is more like observation by a person (in which case it would seem to be privacy-invading) or by a cat (in which case it would seem not to be). We believe that the key difference between the cat and its owner is that the latter, but not the former, is a being capable of “semantic understanding” (Macnish, 2020), or of attaching “meaning which incorporates an element of the observer's reaction” (Soifer and Elliott, 2014). It is only the capacity for an observer to attach meaning to what is observed that constitutes a threat to privacy *per se*. In this way, it seems that AI systems are more like the cat than like its owner, and thus in fact do not pose a threat to privacy *per se*.

Another important aspect to consider when examining privacy in the context of AI is the element of the “feedback loop.” As noted above, people usually have an opportunity to see how their persona was received by others, and adjust both their self-perceptions and their future persona-building attempts accordingly. We claimed that this “feedback loop” has value because of its important connection with autonomy. It may be important to consider, then, whether a similar feedback loop arises in connection with AI data-gathering systems.

The immediate answer seems to be “no.” In the normal operation of AI data-gathering, the system profiles people largely outside of peoples' knowledge and control. Once the data-gathering has been initiated by the system, data subjects rarely—if ever—gain access to the profiling that it has completed. Whatever the system learns, is not something explicitly available for the re-examination or re-consideration of a data subject's awareness, nor their behavior. So, for example, social media accounts may be suspended with no greater reason being given than having “violated terms of service,” or people can be denied loans on the basis of a poor credit rating without ever knowing what it was that brought their credit ratings down. Furthermore, the algorithmic processes involved and the interim conclusions the system comes to or relies on in its data processing can remain obscure, and possibly unknown, for even the computer scientists and software engineers who developed the system itself [There are, of course, a number of interesting ethical issues raised by this “black box” issue (Durán and Jongsma, 2020; Véliz et al., 2021), but exploring them here would take us too far away from the focus of this article]. As such, it seems that “profiling” by AI does not have the same sort of effect on the development of autonomy as perception by one's fellow humans typically does.

It might be argued that there is after all some version of the feedback loop in operation with regard to AI systems, in that people do sometimes have at least some insight into the sorts of things that will generate a profile of a particular type, and know that if they take steps designed to alter this profile, they may be able to see whether their steps are proving effective. For example, people might conscientiously do things specifically to try to build up a good credit rating (e.g., by charging small amounts to a credit card and paying those amounts off promptly). They can also get a sense of whether their efforts to develop a desired sort of profile have been successful or not, and may be able to determine what needs to be remedied to change an unwanted result.

The question arises, however, about how substantial an effect this has on the development of the autonomous self. Some might argue that mass surveillance has a tendency to shape us in subtle but very harmful ways into the sorts of beings that the operators of the technology want us to be (Cohen, 2013). If so, it does seem to participate in the feedback loop to a large enough extent to influence our development as moral agents. It should be noted, however, if there is a feedback loop at all, it does not seem to be of a sort that will enhance autonomy. Indeed, it might be argued that this “shaping” has a tendency to deny people the time and space for reflection necessary for genuine autonomy. Furthermore, even if it does influence our autonomy in some way, this might not be a matter of privacy. Indeed, we have argued that privacy plays an important role in the development of autonomy, but this does not mean that every instance of autonomy is also an instance of privacy. And here, if what one becomes is not guided by a desire to be perceived in a particular way (but only, say, by personal convenience), then it does not seem to be connected with privacy in the way we have outlined at all.

It is also important to note, however, that the feedback loop provided through AI is likely to come up in connection with only a small number of aspects of one's identity, and probably not the most significant ones at that (For example, somebody could reasonably say “I would still be ‘me,' regardless of whether I had a good credit rating or not.”). Certainly our interactions with AI do not have nearly the same sort of influence as our interactions with other humans do, in helping us to understand who we are and what we value.

## Conclusion

Overall, we have seen that AI systems do have impact on privacy in a number of ways, but not always in the ways that people might think they do. AI systems do challenge several features commonly associated with privacy, such as the epistemic privilege that people generally enjoy with regard to information about themselves, and the sort of control over information about one's self that people count on in ordinary interpersonal interactions. However, it is not clear that this, by itself, has much impact on privacy. This is true, we have argued, largely because privacy *per se* is concerned fundamentally with an interest in how other people perceive us, and AI systems do not form the kind of perceptions that can interfere with this interest. We must be aware, however, that the presence of large amounts of information about people within the AI systems does create an increased risk that privacy will be violated, if that information is accessed by a being capable of forming perceptions on the basis of that data. Although AI systems do not constitute as much of a threat as people may think to privacy *per se*, this does not mean there are no dangers associated with them. This becomes clear once one appreciates the distinction between an interest in privacy *per se*, which arises immediately upon other people acquiring information about one, and an interest in security, which can arise when others make use of information gathered about one in ways that damage the person's interests. Being capable of attaching meaning is not required in order to pose a threat to security interests, and there is no need for a being capable of semantic understanding to access the information gathered by AI systems in order for a threat to security interests to be realized. At the same time, we think it is a mistake for people to be concerned only about security interests, and disregard interests in privacy *per se*, since interests in privacy *per se* in fact play an important role in the development of autonomy and personal identity. We believe that improving clarity on these concepts and the relationships between them is very important in being able to explain to people what they do and do not have reason to be concerned about in regard to AI technologies and privacy.

## Data Availability Statement

The original contributions presented in the study are included in the article/supplementary material, further inquiries can be directed to the corresponding author/s.

## Author Contributions

Both authors listed have made a substantial, direct, and intellectual contribution to the work and approved it for publication.

## Conflict of Interest

The authors declare that the research was conducted in the absence of any commercial or financial relationships that could be construed as a potential conflict of interest.

## Publisher's Note

All claims expressed in this article are solely those of the authors and do not necessarily represent those of their affiliated organizations, or those of the publisher, the editors and the reviewers. Any product that may be evaluated in this article, or claim that may be made by its manufacturer, is not guaranteed or endorsed by the publisher.
